# Femoral Arteriovenous Grafts for Hemodialysis: Retrospective Comparison With Upper Extremity Grafts and Fistulas

**DOI:** 10.1177/2054358117719747

**Published:** 2017-07-25

**Authors:** Chance Dumaine, Gabriela Espino-Hernandez, Alexandra Romann, Rick Luscombe, Mercedeh Kiaii

**Affiliations:** 1 Division of Nephrology, Department of Medicine, St. Paul’s Hospital, University of Saskatchewan, Saskatoon, Canada; 2 British Columbia Provincial Renal Agency, Vancouver, Canada; 3 Providence Health Care, Department of Nursing, Vancouver, British Columbia, Canada; 4 Division of Nephrology, Department of Medicine, The University of British Columbia, Vancouver, Canada

**Keywords:** arteriovenous access, arteriovenous fistula, arteriovenous graft, renal dialysis, ESRD, vascular access

## Abstract

**Background:**

Femoral arteriovenous grafts are rarely used to provide vascular access for dialysis patients. This is likely due, in part, to historically high rates of graft loss from infection and thrombosis. However, for selected patients who have exhausted all access options in the upper extremity, femoral grafts can provide additional sites for access creation and may be preferred over central venous catheters.

**Objective:**

We sought to demonstrate that femoral grafts can provide a reliable and safe alternative to central venous catheters for selected patients.

**Methods:**

A single-center retrospective review in Vancouver, Canada, from April 1, 2008, to March 31, 2012, was conducted. All patients with new arteriovenous access (grafts and fistulas) created during the study period were included in the study population and followed for a minimum of 2 years. Comparisons of patency (primary, secondary, and functional) and complications (infectious and noninfectious) were made between the different access types.

**Results:**

Thirteen patients with femoral grafts were compared with 22 patients with arm grafts and 384 patients with fistulas. Femoral grafts had higher rates of thrombosis (46% with a thrombotic event) and a higher requirement for interventions (1.3 angioplasties and 0.12 thrombolytic procedures per patient per year). However, compared with arm grafts, femoral grafts had superior secondary and functional patency. No difference in patency was seen when comparing femoral grafts with upper extremity fistulas. Only 2 patients with femoral grafts required antibiotics for infection, and no grafts were lost to infection.

**Conclusions:**

For patients with limited access options remaining, femoral grafts may provide an additional form of vascular access before resorting to catheter use. Our study shows that with appropriate patient selection, femoral grafts have low infection rates and patency that is comparable with other access types.

## Abrégé

**Contexte:**

De manière générale, les greffons artérioveineux fémoraux sont rarement utilisés pour fournir des accès vasculaires aux patients dialysés, très probablement en raison des taux historiquement élevés de perte du greffon due à une infection ou à une thrombose. Toutefois, pour certains patients ayant épuisé toutes les options d’accès dans les membres supérieurs, les greffons fémoraux peuvent fournir des sites supplémentaires pour la création d’un accès vasculaire et peuvent être préférés aux cathéters veineux centraux.

**Objectif de l’étude:**

Nous avons voulu démontrer que les greffons fémoraux peuvent fournir une solution de rechange fiable et sûre aux cathéters veineux centraux chez certains patients.

**Méthodologie:**

Il s’agit d’une étude rétrospective qui s’est tenue dans un centre hospitalier de Vancouver, au Canada, entre le 1er avril 2008 et le 31 mars 2012. Tous les patients chez qui on a procédé à un nouvel accès artérioveineux (greffons ou fistules) au cours de la période d’étude ont été inclus. Les patients recrutés ont été suivis sur une période minimale de deux ans. La perméabilité vasculaire (primaire, secondaire et fonctionnelle) et les complications rapportées (infectieuses et non infectieuses) ont été comparées entre les différents types d’accès.

**Résultats:**

Pour cette étude, treize patients avec greffons artérioveineux fémoraux ont été comparés à 22 patients avec greffons artérioveineux brachiaux et 384 patients avec fistules. Les greffons fémoraux ont présenté des taux plus élevés de thrombose (46% des patients ont subi un événement thrombotique) et nécessité davantage d’interventions (moyenne de 1,3 angioplastie et de 0,12 procédure thrombolytique par patient par année). Toutefois, lorsque comparés aux greffons brachiaux, les greffons fémoraux présentaient des valeurs de perméabilité secondaire et fonctionnelle supérieures. Aucune différence de perméabilité n’a cependant été observée lors de la comparaison des greffons fémoraux et des fistules des membres supérieurs. Seuls deux patients avec un greffon artérioveineux fémoral ont dû être traités aux antibiotiques pour soigner une infection, et aucune perte de greffon en raison d’une infection n’a été observée au cours de l’étude.

**Conclusions:**

Pour les patients dont les possibilités d’accès vasculaire sont limitées, le recours à un greffon artérioveineux fémoral peut s’avérer une option supplémentaire avant de devoir recourir à une sonde. Notre étude montre qu’en portant une attention particulière à la sélection des patients, les greffons artérioveineux fémoraux présentent de faibles taux d’infections et une perméabilité comparable à celle des autres types d’accès vasculaires.

## What was known before

If creation of an upper extremity graft or fistula is not an option, most clinicians will opt for a central venous catheter rather than create arteriovenous access in the lower extremity.

## What this adds

Our retrospective review of data from a large, tertiary care center in Canada (St. Paul’s Hospital, Vancouver) shows that femoral grafts have excellent patency and very few infectious complications in carefully selected patients.

## Introduction

Current guidelines promote the use of upper extremity arteriovenous fistulas (AVFs) as the preferred vascular access for patients on hemodialysis.^1^ If patient anatomy or other conditions prohibit the creation of a fistula, prosthetic arteriovenous grafts (AVGs) are the preferred second choice, followed by central venous catheters (CVCs). When the vasculature of the upper limb has been exhausted, the lower extremity can be used for AVF creation (typically through femoral vein transposition) or AVG placement; both options have been shown to have acceptable patency rates and infectious complications in appropriately selected patients.^2-7^ However, many clinicians still opt to place a tunneled CVC rather than utilize femoral access. Factors contributing to this decision are multifactorial but may relate to inexperience with the creation or management of femoral grafts and fistulas. In addition, concerns regarding access survival and infectious complications may deter some clinicians from attempting femoral access.

At St. Paul’s Hospital in Vancouver, British Columbia, Canada, we frequently assess patients for femoral graft placement when there are no remaining options for arteriovenous access in the upper extremity. Our dialysis unit uses a computerized database to document vascular access creation, complications, interventions, and survival. This has allowed us to conduct a retrospective review of our femoral graft use and compare outcomes with upper extremity AVFs/AVGs (the preferred methods of vascular access).

## Methods

The dialysis unit at St. Paul’s Hospital in Vancouver, Canada, provides chronic dialysis to approximately 250 prevalent hemodialysis patients per year. All patients requiring vascular access creation are assessed in a multidisciplinary vascular access clinic, which consists of a vascular surgeon, nephrologist, and vascular access nurse. Ultrasound vein mapping is performed in the clinic, and a team-based decision is made regarding the optimal access for each patient. As per current clinical practice guidelines, preference is given to creation of an upper extremity AVF whenever possible. (During our data collection period, 62% of dialysis patients at our center utilized AVFs, 7% utilized AVGs, and 31% utilized CVCs as their primary dialysis access).

An AVG is considered for patients who have either exhausted their arm vessels for AVFs, who do not have adequate vasculature for AVFs, or who have evidence of extensive arterial disease that would increase their risk of steal syndrome. Forearm grafts are rarely created at our center. Upper arm AVGs are considered an option if there is an adequately sized outflow vein (usually the axillary vein) for graft-vein anastomosis, no evidence of central vein stenosis, and no arterial disease to suggest increased risk of steal. Patients who have chronically low blood pressure are deemed unsuitable for arm grafts at our center, as our previous experience has shown that such patients often experience recurrent graft thrombosis.

In cases where neither an upper extremity AVF or AVG are deemed suitable, femoral graft placement is considered. Patients are screened for lower extremity arterial disease by physical examination from a vascular surgeon. Investigations including ultrasound to assess deep vein patency, computed tomography angiogram with arterial and venous phases, and toe pressures are often performed at the discretion of the vascular access clinic. Patients thought to have moderate or severe peripheral vascular disease and high risk of distal ischemia after graft creation are ineligible for femoral AVG. For patients who are eligible for femoral AVG, a polytetrafluoroethylene graft is placed in a loop configuration between the superficial femoral artery and the common femoral vein at the saphenofemoral junction. While lower extremity AVF creation via femoral vein transposition is performed at some centers, this is not routinely done in Vancouver due to limited surgical experience and a high rate of ischemic complications with the small number performed in the past.

Once patients initiate hemodialysis, access flows are measured every 4 to 6 weeks by ultrasound dilution technique. Patients are referred for angiogram if there are clinical indicators of stenosis (difficulty needling, difficulty achieving hemostasis, high venous pressures, etc) or if there is a more than 25% reduction in access flow from baseline.

### Patient Selection

Ethical approval was obtained from the Providence Health Care Research Ethics Board. Patient and access data were extracted from the Provincial Record and Outcome Management Information System (PROMIS), which stores prospectively collected data from all patients referred for kidney disease in British Columbia, Canada. This database is managed by the British Columbia Provincial Renal Agency, and all vascular access data are entered into PROMIS in real time. All patients who underwent AVG placement (both upper extremity and lower extremity) or upper extremity AVF creation between April 1, 2008, and March 31, 2012, at St. Paul’s Hospital were identified as study participants. Exclusions were made for patients who died, received a transplant, transitioned to peritoneal dialysis, or stopped hemodialysis for another reason within 3 months of access creation.

### Data Collection

Follow-up data were collected until March 31, 2014, to ensure a minimum follow-up of 2 years. Data obtained included baseline demographic information (gender, age, and ethnicity), medical comorbidities (including diabetes, cardiovascular disease, and peripheral vascular disease), and data on the access (date of creation, infectious and noninfectious complications, number and efficacy of interventions, and date of access failure).

To allow for comparison, patients were stratified into 6 groups: femoral graft (femoral AVG), arm graft (arm AVG), brachiobasilic AVF (BBF), brachiocephalic AVF (BCF), traditional radiocephalic AVF (RCF), and snuffbox radiocephalic AVF (SBF). The primary outcome examined was access patency. For this, various definitions previously defined in the literature were used.^8^ *Primary patency* was defined as the interval from the time of access placement until any intervention designed to maintain or reestablish patency. *Secondary patency* was defined as the interval from the time of access placement until access abandonment, thrombosis, or the time of patency measurement including intervening manipulations (surgical or endovascular interventions) designed to reestablish functionality in a thrombosed access. In addition to these established definitions, we also studied functional patency, which we defined as the interval from the time of first use of the access for hemodialysis until access abandonment or thrombosis.

Secondary outcomes included rates of primarily failure (failure of the access without ever being used, either from failure to mature or thrombosis prior to first use), access infection requiring systemic antibiotics/access removal, thrombotic events, noninfectious complications (including aneurysm, pseudoaneurysm, severe limb edema, steal syndrome, and access breakdown), and requirement for intervention to maintain patency.

### Statistical Analysis

For each of the 6 groups, continuous variables in the study were presented as median (interquartile range) and categorical variables were displayed as count (frequency). To assess differences with respect to the femoral AVG group, data were compared using 2-sided Wilcoxon rank-sum test for continuous variables and 2-sided Fisher exact test for categorical variables, with *P* < .05 considered statistically significant. Patency rates were estimated using Kaplan-Meier analysis and truncating data at 1 and 2 years. Primary failures were excluded from 1- and 2-year patency calculations. Kaplan-Meier curves of each group were compared with the Kaplan-Meier curve of the AVG leg group for each of the time periods using the log-rank test. Analyses were performed using SAS Version 9.3 (SAS Institute, Cary, North Carolina), and figures were generated using R 3.3.2 (R Core Team 2016, Vienna, Austria).

## Results

A total of 419 patients with either AVG or AVF were included in data analysis. Baseline characteristics for each group are shown in Table 1.

**Table 1.** Baseline Characteristics by Access Type.

Variables	Overall	AVG		AVF			
		Leg	Arm	BBF	BCF	RCF	SBF
Total (patients)	419	13 (3.1%)	22 (5.3%)	87 (20.8%)	173 (41.3%)	69 (16.5%)	55 (13.1%)
Age, y	69 (57-77)	74 (57-76)	73 (59-83)	69 (55-78)	70 (58-77)	67 (57-75)	67 (51-79)
Gender: male	267 (63.7%)	3 (23.1%)	12 (54.5%)	57 (65.5%)	107 (61.8%)	51 (73.9%)	37 (67.3%)
Race							
Caucasian	177 (42.2%)	7 (53.8%)	7 (31.8%)	39 (44.8%)	75 (43.4%)	23 (33.3%)	26 (47.3%)
Asian Filipino	65 (15.5%)	0	2 (9.1%)	4 (4.6%)	55 (31.8%)	3 (4.3%)	1 (1.8%)
Asian Oriental	92 (22%)	4 (30.8%)	7 (31.8%)	22 (25.3%)	13 (7.5%)	26 (37.7%)	20 (36.4%)
Other	74 (17.7%)	2 (15.4%)	6 (27.3%)	21 (24.1%)	24 (13.9%)	15 (21.7%)	6 (10.9%)
Missing/Unknown	11 (2.6%)	0	0	1 (1.1%)	6 (3.5%)	2 (2.9%)	2 (3.6%)
Comorbidities							
DM	228 (54.4%)	8 (61.5%)	12 (54.5%)	46 (52.9%)	98 (56.6%)	35 (50.7%)	29 (52.7%)
CVD	224 (53.5%)	11 (84.6%)	13 (59.1%)	51 (58.6%)	95 (54.9%)	30 (43.5%)	24 (43.6%)
PVD	57 (13.6%)	1 (7.7%)	4 (18.2%)	9 (10.3%)	28 (16.2%)	7 (10.1%)	8 (14.5%)
Access created before dialysis	173 (41.3%)	0	2 (9.1%)	28 (32.2%)	76 (43.9%)	31 (44.9%)	36 (65.5%)
Dialysis vintage, mo	14 (4-44)	67 (22-112)	24 (13-102)	25 (7-51)	9 (4-37)	5 (3-29)	6 (2-18)

*Note.* Baseline characteristics of patients shown as overall cohort and by access type. Dialysis vintage refers to the duration (in months) on hemodialysis and/or peritoneal dialysis prior to access creation. AVG = arteriovenous graft; AVF = arteriovenous fistula; BBF = brachiobasilic fistula; BCF = brachiocephalic fistula; RCF = radiocephalic fistula; SBF = snuffbox radiocephalic fistula; DM = diabetes mellitus; CVD = cardiovascular disease; PVD = peripheral vascular disease.

A total of 13 patients had a femoral graft placed. There were 22 arm grafts and a total of 384 upper extremity AVFs. The mean age for the overall cohort was 69 years. There were no significant differences in age between femoral AVG and any other group. In all, 63.7% of the study cohort was male. Only 23.1% of the patients with femoral AVG were male, significantly lower than all AVF groups, but not significantly lower than the arm AVG group. In all, 42.2% of the cohort was Caucasian, with no significant difference between the femoral AVG group and any other group.

The femoral graft group had lower rates of peripheral vascular disease than other groups, but the difference was not statistically significant. Approximately half of the study cohort had diabetes, with no significant differences between groups. A large proportion of patients with femoral AVG (84.6%) had documented cardiovascular disease (significantly higher than the BCF, RCF, and SBF groups).

No patients with femoral grafts had their access created before dialysis. This was compared with 9.1% of arm AVG and 44.5% of fistulas (171 of 384) that were created before dialysis. Patients with femoral grafts had been on dialysis for a mean of 67 months before graft placement, a duration that was significantly higher than that of all fistula types.

**Figure 1.**
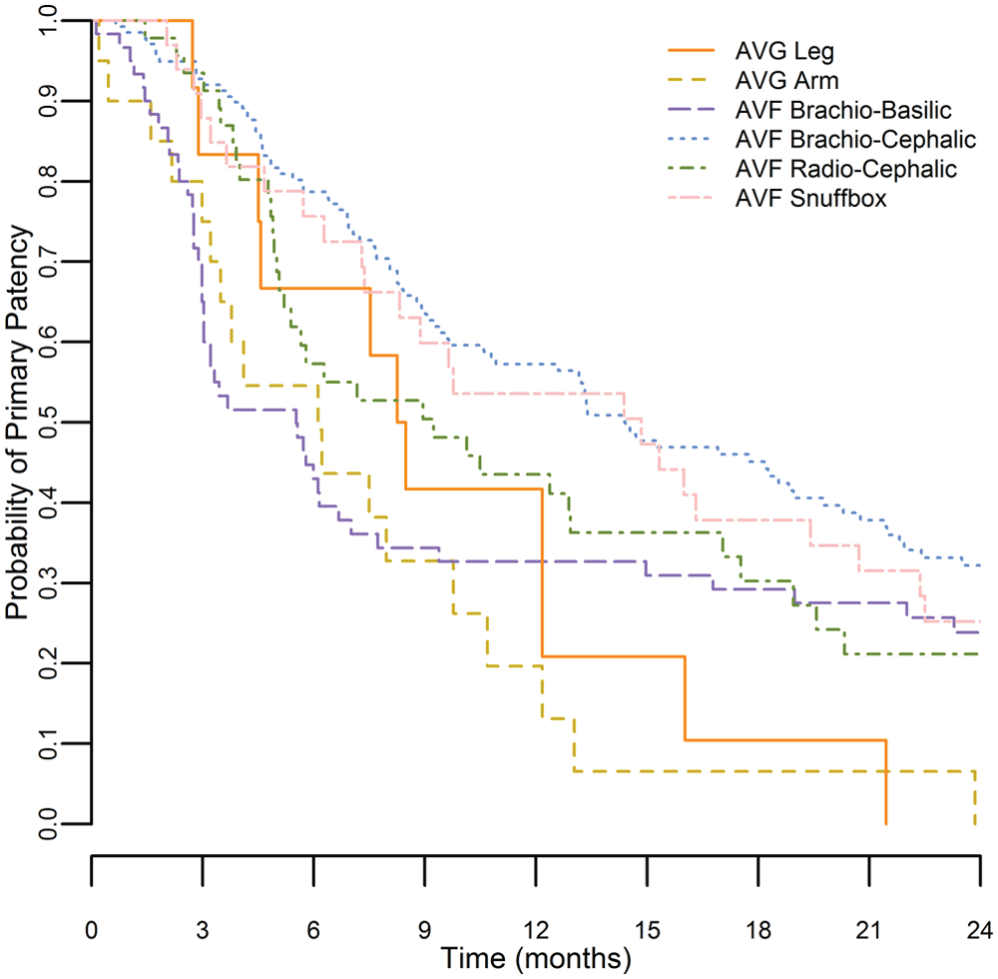
Kaplan-Meier curves for primary patency. *Note.* Kaplan-Meier survival curve is shown for primary patency (time from access creation to first intervention to maintain or reestablish patency) for all access types. AVG = arteriovenous graft; AVF = arteriovenous fistula.

**Figure 2.**
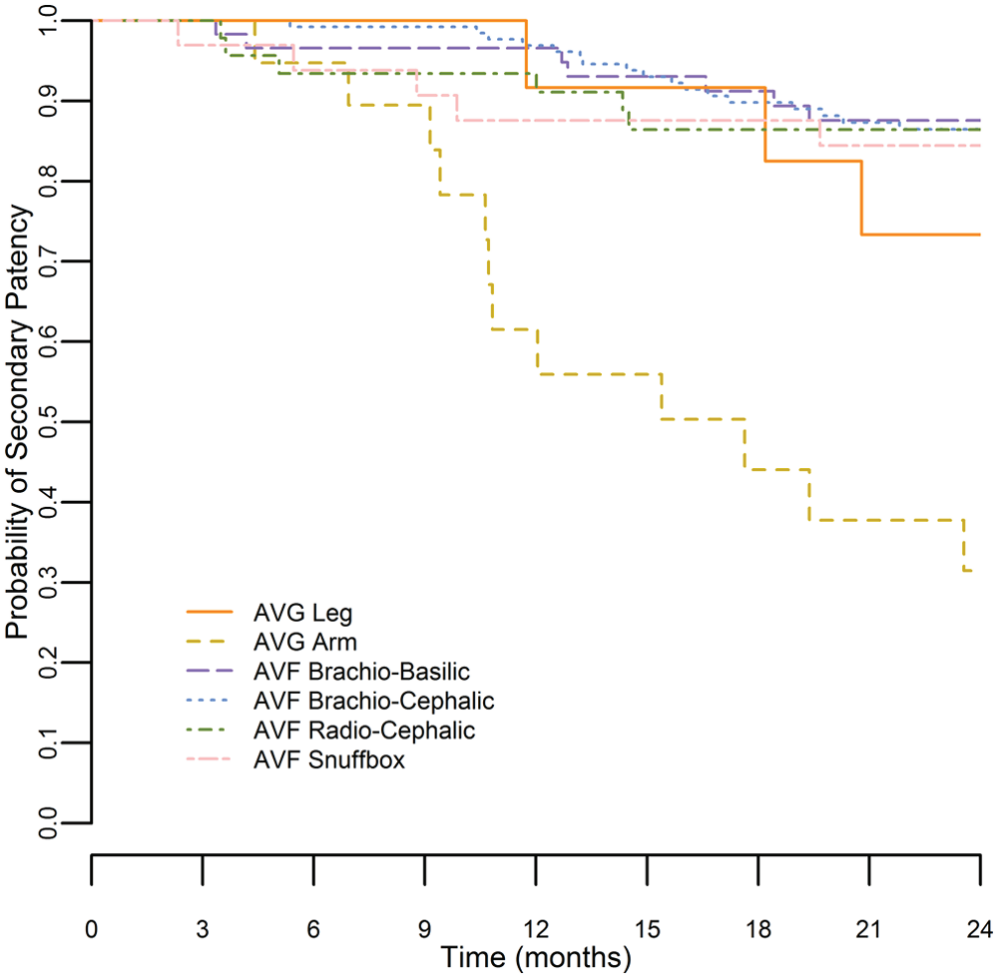
Kaplan-Meier curves for secondary patency. *Note.* Kaplan-Meier survival curve is shown for secondary patency (time from access creation to loss of access from abandonment or thrombosis) for all access types. AVG = arteriovenous graft; AVF = arteriovenous fistula.

**Figure 3.**
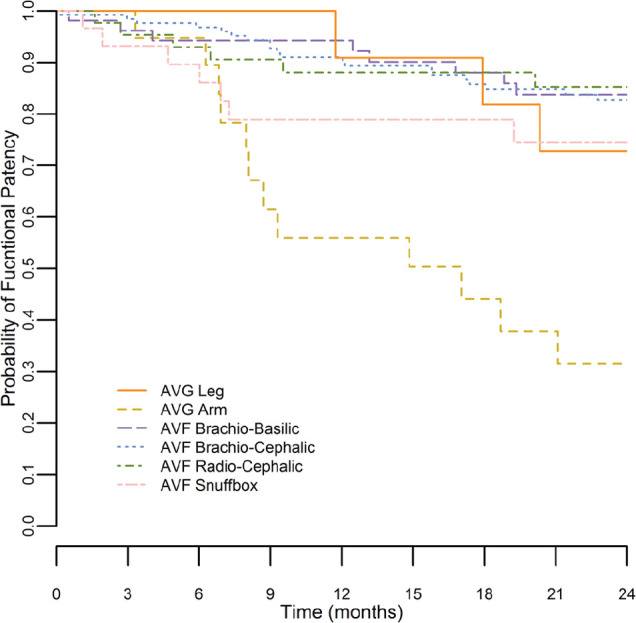
Kaplan-Meier curves for functional patency. *Note.* Kaplan-Meier survival curve is shown for functional patency (time from first use of the access to loss of access from abandonment or thrombosis) for all access types. AVG = arteriovenous graft; AVF = arteriovenous fistula.

Figures 1 to 3 show Kaplan-Meier survival curves for primary, secondary, and functional patency of each graft type. Log-rank testing was used to compare patency curves for femoral grafts with patency curves of other access types.

Primary patency for femoral grafts at 1 year was 42%. One-year primary patency rates were similar for all other access types (20% for arm grafts and 33% to 57% for fistulas) with no significant differences noted. Two-year primary patency rates were low for all access types. No grafts (femoral or arm) survived 2 years without intervention.

Secondary patency for femoral grafts was 92% at 1 year. One-year secondary patency for arm grafts was not significantly different (62%, log-rank *P* value = .35). One-year secondary patency for AVFs ranged from 88% to 97% depending on fistula type; when comparing survival curves, there was no difference between femoral AVGs and any type of AVF.

The secondary patency at 2 years was superior for femoral grafts (73%) than for arm grafts (31%, log-rank *P* value = .02). Secondary patency for fistulas at 2 years was 84% to 88% depending on the fistula type. No fistula type was significantly superior to femoral grafts upon comparison of Kaplan-Meier survival curves.

Functional patency for femoral AVG was 91% at 1 year and 73% at 2 years. Functional patency for femoral AVG was significantly better than arm AVG at both 1 and 2 years (91% vs 56% [log-rank *P* value = .03] and 73% vs 31% [log-rank *P* value = .02], respectively). No significant differences were seen in functional patency between femoral AVG and any type of AVF at either 1 or 2 years.

Table 2 outlines the various secondary outcomes. Of the 13 femoral grafts created, only 1 had primary failure (7.7%) compared with 9.1% of arm grafts. All fistulas had much higher rates of primary failure, ranging from 13.9% for brachiocephalic fistula to 29.1% for snuffbox fistula.

**Table 2.** Secondary Outcomes by Access Type.

	Overall	AVG		AVF			
		Femoral	Arm	BBF	BCF	RCF	SBF
Total	419	13	22	87	173	69	55
At least one angioplasty	171 (40.8%)	9 (69.2%)	12 (54.5%)	13 (14.9%)	79 (45.7%)	34 (49.3%)	24 (43.6%)
Thrombotic event	93 (22.2%)	6 (46.2%)	12 (54.5%)	21 (24.1%)	27 (15.6%)	16 (23.2%)	11 (20%)
Other noninfectious complications	93 (22.2%)	5 (38.5%)	10 (45.5%)	24 (27.6%)	44 (25.4%)	4 (5.8%)	6 (10.9%)
Primary failure	83 (19.8%)	1 (7.7%)	2 (9.1%)	22 (25.3%)	24 (13.9%)	18 (26.1%)	16 (29.1%)

*Note.* “Other noninfectious complications” include aneurysm, pseudoaneurysm, edema, rupture, steal syndrome, and access breakdown. AVG = arteriovenous graft; AVF = arteriovenous fistula; BBF = brachiobasilic fistula; BCF = brachiocephalic fistula; RCF = radiocephalic fistula; SBF = snuffbox radiocephalic fistula.

Both femoral and arm grafts had higher rates of thrombosis compared with fistulas. The only significant difference was between femoral grafts and brachiocephalic fistulas (46.2% vs 15.6%, *P* = .01). Other noninfectious complications were also higher in both femoral and arm grafts than in fistulas (Table 2).

Requirement for intervention was calculated as mean number per patient per year. Femoral grafts required 1.31 angioplasties per year, less than arm grafts (2.2 per year) but more than any fistula type (0.61-1.08 per year). Thrombolytic/thrombectomy requirement was 0.12 per patient per year for leg grafts. This was less than arm grafts (0.2 per year) but more than any fistula type (0-0.02 per year).

Two patients with a leg graft (15.4%) required systemic antibiotics for infection. No leg grafts had to be surgically removed because of infection.

## Discussion

Our review has shown that at our center, femoral grafts have excellent outcomes. Although primary patency (patency without intervention) at 1 year was low (42%) and no femoral grafts survived to 2 years without intervention, secondary patency (patency assisted by intervention) was impressive at both 1 year (92%) and 2 years (73%).

Current clinical practice guidelines promote the creation of fistulas in the upper extremity as the preferred form of vascular access for long-term hemodialysis.^1^ When circumstances do not permit AVF creation, arm grafts are preferred over CVCs because observational studies have shown lower rates of infection and better patient survival.^1,4,10,11^ In addition, grafts avoid other complications that may occur with CVC use, such as central venous stenosis. However, when upper extremity anatomy is unsuitable for fistula creation, creating a graft in the arm is often not possible either.

Despite literature showing that femoral access can be safely created and used in selected patients, most patients without access options in the arm have tunneled dialysis catheters. This decision is likely multifactorial and may relate to limited surgical experience with femoral AV access creation and concerns of infection due to the anatomical location of the access near the groin. Some clinicians may also be wary of femoral grafts because of historically high rates of graft loss due to thrombosis; previous studies have shown poor survival of femoral grafts, with some studies showing more than 50% of grafts being lost by 1 year.^12,13^ However, in the current era of interventional radiology, early graft loss from thrombosis poses less of a concern, as experience has shown grafts are often salvageable with endovascular treatment for days after thrombosis. In this study, we sought to show that femoral AVGs are a viable option for appropriately selected patients who have exhausted their upper extremity vasculature.

In our cohort, the secondary and functional patency of femoral grafts was superior to arm grafts and was no different than fistulas. This is in keeping with previous studies that have documented that femoral grafts have similar (or even better) patency rates than other forms of access. A single-center review by Miller et al found that arm and leg grafts had similar secondary survival.^2^ Subsequently, a 2010 review by Ram et al showed leg grafts had better cumulative survival than both arm grafts and fistulas up to 5 years after creation.^5^

Other studies have documented 5-year femoral graft patency of 47% to 54%.^5,6,14^ Although we do not have 5-year data on our study cohort, our 2-year secondary survival was similar to that reported in each of those studies; we therefore would expect similar 5-year survival rates for femoral grafts at our center.

Our data show that femoral grafts had higher rates of thrombosis than any fistula type. However, secondary and functional patency rates were no different than that of fistulas, suggesting that thrombosed femoral grafts can be effectively salvaged with timely intervention. To maintain a secondary patency of 73% at 2 years, an average of 1.44 interventions (angioplasties and thrombolysis) per patient per year was required for patients with femoral grafts. This was higher than for patients with fistulas (0.63-1.08 interventions per patient per year depending on fistula location) but much less than for patients with arm grafts (2.4 interventions per patient per year). For patients with limited access options remaining, this seems to be a reasonable number of interventions to maintain them on hemodialysis.

As previously mentioned, many clinicians opt for tunneled CVC rather than femoral grafts once access options in the arm have run out. A major factor in this decision may be fear of infection in femoral grafts. Ong et al evaluated long-term outcomes of leg grafts compared with tunneled internal jugular catheters and found leg grafts to be superior from both a patency and infectious perspective.^4^ At 5 years, 38% of leg grafts were still functional, but no catheters survived longer than 900 days. At 5 years, 61% of leg grafts remained infection free; no catheters remained infection free for longer than 2 years. In keeping with that data, we had very few infectious complications in femoral grafts at our center. Only 2 of the 13 grafts that were placed required antibiotics for local infection. In both cases, the infection was controlled with antibiotics and graft excision was not required. This may be a reflection of the relatively short follow-up period in our study. However, a previous single-center study has shown only 14% of leg grafts created over a 9-year period required surgical excision secondary to infection.^15^

The willingness of patients to accept femoral grafts and the impact of femoral grafts on patient quality of life are not well described in the literature and are areas that deserve further study. Anecdotally, patients at our center who have received femoral grafts have not expressed any regrets following access creation; however, some patients who refuse femoral grafts cite concerns regarding the appearance of the graft and potential interference with sexual activity. These concerns should be addressed by the health care team when making decisions regarding vascular access.

Finally, while we believe that femoral grafts are an appropriate alternative to CVCs for some patients, we recognize that femoral grafts are not suitable for all patients. For patients with limited life expectancy, CVC insertion and avoidance of further surgical procedures may be a more appropriate option than a femoral graft. Other patient factors, such as poor personal hygiene, may also make patients poor candidates for femoral access. Although the literature shows low rates of infection with femoral access, we expect that many centers (including ours) use patient hygiene as an informal screen to determine eligibility. The ability to keep the access site clean is of utmost importance for any access type, including femoral grafts.

## Limitations

Despite promising results showing that femoral grafts can provide adequate vascular access, there are a number of limitations to our study. The primary limitation is a relatively small number of femoral grafts in our dialysis population compared with that from previous studies. Our center attempts upper extremity fistula creation whenever possible and only explores lower extremity access when all upper extremity options have been utilized. Over the 5-year period studied, only 13 femoral grafts were created. However, to the best of our knowledge, this is the only Canadian publication to date outlining success rates of femoral grafts compared with other forms of vascular access. The small number of femoral grafts in our center is likely comparable with the majority of other Canadian hemodialysis centers.

A further limitation to our study is that as a retrospective review, there is the potential that not all complications or access failures were properly recorded, despite a rigorously maintained prospective database in our dialysis unit. In addition, there were no standardized selection criteria for femoral graft creation. Patients were selected after a multidisciplinary assessment at our vascular access clinic. Proper selection of patients is undoubtedly critical to ensuring graft success; however, as the decision to create femoral grafts was not standardized, there is the potential for selection bias in our review, and centers using different selection criteria may not have similar results.

Finally, the generalizability of our results may be limited by a number of factors. Successful creation and maintenance of femoral grafts requires dedication from both patients and vascular access clinicians. The patients at our center who receive femoral grafts represent a subset of patients who are motivated to avoid CVC insertion and who are willing to undertake extra surgical procedures to do so. It is expected that many patients with no remaining options for arteriovenous access in the upper extremity will opt for CVC insertion rather than femoral access; this is a decision that should be made in conjunction with their health care team. The success of femoral grafts in our patients is also due to the efforts of our multidisciplinary vascular access team. Centers without dedicated vascular access resources to properly maintain femoral grafts may not have the same success rate we have had at St. Paul’s Hospital. Finally, a large proportion of our dialysis population is of Asian descent, and it may be difficult to generalize our results to dialysis populations of different ethnic backgrounds.

## Conclusions

In patients with no further options for vascular access in the upper extremity, clinicians must make a decision between pursuing arteriovenous access in the leg versus placement of a tunneled dialysis catheter. In our experience, femoral grafts can provide high-quality vascular access for selected patients, with short-term patency and infectious complications comparable with upper limb fistulas and grafts. Graft loss secondary to infection did not occur in our study, and previous studies have shown that infectious complications are less common in femoral grafts than in dialysis catheters. The literature has also shown that long-term patency of femoral grafts is superior to dialysis catheters.

Experience from other centers would suggest that long-term patency could be achieved in a large number of femoral grafts with ongoing monitoring and intervention. A caveat to this is that femoral grafts require frequent interventions to maintain patency; timely access to interventional radiology should be a consideration for any centers considering femoral graft creation.

Although we still advocate that fistulas should be utilized as the primary access for eligible patients, we believe femoral grafts may be a better option than CVCs in properly selected patients. Further study is needed to see whether similar outcomes with femoral grafts can be achieved at other centers.
